# Stain-free identification of tissue pathology using a generative adversarial network to infer nanomechanical signatures†

**DOI:** 10.1039/d1na00527h

**Published:** 2021-09-02

**Authors:** Lydia Neary-Zajiczek, Clara Essmann, Anita Rau, Sophia Bano, Neil Clancy, Marnix Jansen, Lauren Heptinstall, Elena Miranda, Amir Gander, Vijay Pawar, Delmiro Fernandez-Reyes, Michael Shaw, Brian Davidson, Danail Stoyanov

**Affiliations:** Wellcome/EPSRC Centre for Surgical and Interventional Sciences London W1W 7TS UK lydia.zajiczek.17@ucl.ac.uk; Department of Computer Science, University College London London WC1E 6BT UK; Department of Medical Physics and Biomedical Engineering, University College London London WC1E 6BT UK; Department of Pathology, UCL Cancer Institute, University College London London WC1E 6BT UK; Department of Cellular Pathology, Royal Free Hospital London NW3 2QG UK; Biobank and Pathology Translational Technology Platform, UCL Cancer Institute, University College London London WC1E 6BT UK; Department of Surgical Biotechnology, University College London London WC1E 6BT UK; National Physical Laboratory Teddington TW11 0LW UK

## Abstract

Intraoperative frozen section analysis can be used to improve the accuracy of tumour margin estimation during cancer resection surgery through rapid processing and pathological assessment of excised tissue. Its applicability is limited in some cases due to the additional risks associated with prolonged surgery, largely from the time-consuming staining procedure. Our work uses a measurable property of bulk tissue to bypass the staining process: as tumour cells proliferate, they influence the surrounding extra-cellular matrix, and the resulting change in elastic modulus provides a signature of the underlying pathology. In this work we accurately localise atomic force microscopy measurements of human liver tissue samples and train a generative adversarial network to infer elastic modulus from low-resolution images of unstained tissue sections. Pathology is predicted through unsupervised clustering of parameters characterizing the distributions of inferred values, achieving 89% accuracy for all samples based on the nominal assessment (*n* = 28), and 95% for samples that have been validated by two independent pathologists through *post hoc* staining (*n* = 20). Our results demonstrate that this technique could increase the feasibility of intraoperative frozen section analysis for use during resection surgery and improve patient outcomes.

## Abstract 2

Sample-wide elastic modulus is inferred from unstained images of frozen liver tissue sections. Distribution parameters can predict tissue pathology for use as an intraoperative diagnostic tool.

## Graphical abstract

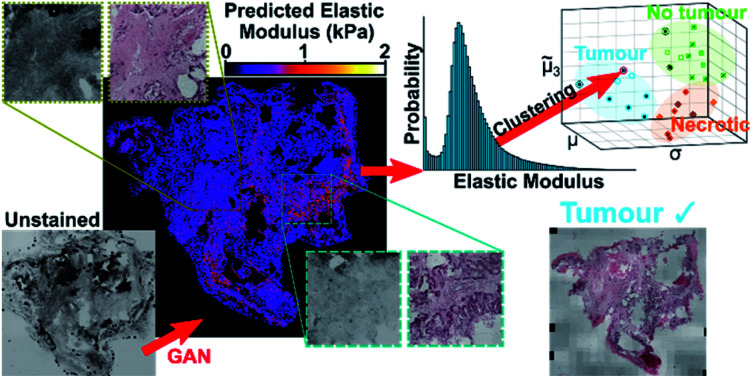

## Introduction

Surgical removal is the gold standard treatment option for many solid cancers and is often the only curative option. Its overall effectiveness relies on accurate estimation of the spatial extent of the cancer to ensure it is completely removed while avoiding unnecessary excision of healthy tissue. Intraoperative frozen section (IFS) analysis can increase tumour margin estimation accuracy through histopathological assessment of stained tissue while the patient remains on the operating table. IFS is used in many types of cancer resection surgeries, including colorectal liver metastases,^1^ lung,^2^ uterine,^3^ ovarian^4^ and breast,^5^ however debate continues on the usefulness of the procedure in other surgeries such as pancreatectomies^6^ and gastric resection.^7^ In cases of basal cell carcinoma, IFS enabled surgeons to conserve tissue while confirming clearance of excision margins;^8^ while in prostatectomies, IFS can be used to determine whether a nerve sparing procedure can be carried out, which greatly improves post-procedure quality of life for patients in maintaining continence and sexual function.^9^

The drawbacks of IFS include the extra time the patient spends under anaesthetic while the tissue is stained and analyzed, increasing their risk, as well as the additional resources required. One trial using IFS during prostatectomies reported that the time from specimen removal to pathology reporting varied from 18 to 47 minutes.^10^ IFS is also less accurate compared with formalin-fixed paraffin-embedded (FFPE) histopathology due to freezing artefacts and difficulties obtaining tissue sections of sufficient quality.^11^ Gagné *et al.* concluded that the additional information gained was not worth the added time and expense in the case of lung cancer resection,^12^ while a study of intraoperative breast margin assessment techniques found that despite the benefits of IFS, the cost and turnaround time was a significant barrier to widespread adoption.^13^ The main contributing factor to each of these limitations is the staining and assessment process: the sample is snap frozen and transported to a pathology laboratory where multiple sections must be cut, fixed, stained and assessed by a pathologist to determine if tumour cells are present at the excision margin, requiring further tissue removal.

A number of novel imaging modalities have been proposed to streamline this process. One uses a much simpler stain combined with confocal fluorescence and reflectance contrast microscopy, which allows for imaging of thicker tissue due to its optical sectioning ability. The resulting pseudo-color image mimics a haematoxylin and eosin (H&E) stain and is assessed by a pathologist as in the standard procedure.^14^ Another technique uses stimulated Raman spectroscopy (sometimes in tandem with second harmonic generation imaging) to replicate the H&E stain, which has the additional advantage of being label-free.^15,16^ Hollon *et al.* combined this modality with a deep learning classification algorithm for intra-operative diagnosis of brain tumours without pathologist intervention.^17^ However, both of these methods fundamentally rely on a pathological assessment, either made by an expert human or an equivalent algorithm. The former is becoming an increasingly scarce resource^18^ while the latter have found limited clinical deployment due to their brittle nature, where nominally successful algorithms may not maintain diagnostic accuracy when deployed on images outside of their training datasets.^19^

An alternative to image-based pathological assessment uses measurable physical properties of bulk tissue for diagnosis without the need for staining or assessment, avoiding the issue of interobserver variability in pathological diagnoses in general,^20,21^ which can be exacerbated by the image quality issues inherent to IFS.^22,23^ A well-known property of cancerous tissue is its markedly different stiffness relative to surrounding healthy tissue; prior to the advent of laparoscopic and endoscopic surgeries, surgeons often used manual palpation to estimate the extent of a cancerous mass.^24,25^ Localised changes in stiffness can be quantified by measuring the elastic modulus (EM) of the tissue, defined as resistance to elastic deformation under the application of stress.^26^ Single cells cultured from cancerous tissue generally have lower EM (larger deformability) when compared to healthy cells as measured *via* atomic force microscopy (AFM),^27,28^ and this increased cell motility is hypothesized to drive the metastasis of cancerous tissue. The relationship between the EM of bulk tissue and tumour progression is more complex however due to the interaction between cancerous cells and the extra-cellular matrix (ECM).^29–31^ An excellent review of the use of AFM in characterizing localised changes in the EM of various tissue types by Stylianou *et al.* finds that the EM of cancerous tissue is starkly heterogeneous, leading to investigations into the use of these nanomechanical signatures for label-free cancer diagnostics.^32^

**Fig. 1:**
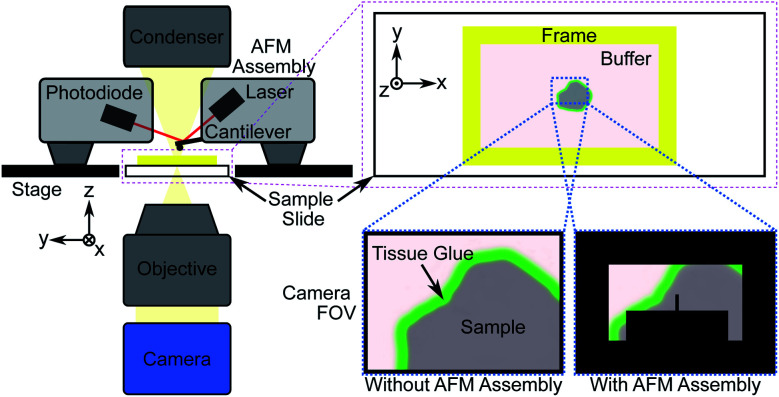
Schematic showing atomic force microscope (AFM) measurement procedure of tissue sections adhered to a glass slide. The cantilever, with a known spring constant, is used to indent a sample and the resulting deflection of the laser beam due to the tissue's structure is measured *via* the photodiode. The measured force and indentation distance are used to compute the elastic modulus (EM) of the tissue.

**Fig. 2:**
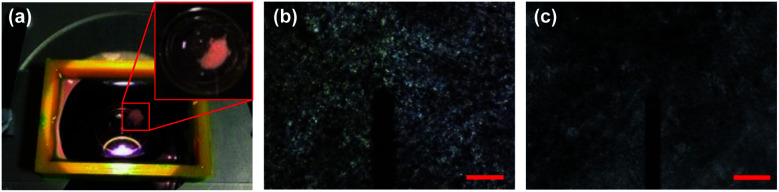
(a) Microscope slide with 3D-printed structure to contain physiological buffer during AFM measurement. Inset: adhered tissue section. (b) AFM microscope field-of-view (FOV) showing 100 μm thick section captured with standard AFM camera at 10× magnification with cantilever visible. Scale bar 100 μm. (c) Identical image in (b) of 40 μm thick section captured with high dynamic range sCMOS camera.

Direct measurement of tissue samples using an AFM is not a feasible replacement for histopathological assessment in an intraoperative setting, however. Sample preparation is non-trivial and measurements are time consuming; measuring a 100 μm^2^ area takes approximately 10 minutes, while single tissue sections can be up to 100 mm^2^ in size, and multiple sections may require assessment for accurate margin estimation. During the limited time period of sample viability after thawing, the number of AFM measurements that can be made will likely not be sufficient to fully characterize a sample, particularly under the time constraints of an intraoperative procedure. An additional limitation is the difficulty in spatially localising such small measurement areas, which is crucial for assessing surgical margins. Previous studies that identified the nanomechanical signatures of disease did so through a large number of “blind” AFM measurements of bulk (≥1 mm thick) tissue sections, statistically correlating the findings with *post hoc* histopathology.^27,33–35^Fig. 1 shows the general procedure for measuring tissue samples using an AFM; the cantilever assembly occludes much of the camera's field-of-view (FOV), reducing the contrast of the resulting image. Even for a 100 μm-thick tissue section as shown in Fig. 2a and b, contrast is poor and accurate spatial localisation of the measurement area is challenging due to the highly scattering nature of the tissue and the resulting lack of recognizable features.

**Fig. 3:**
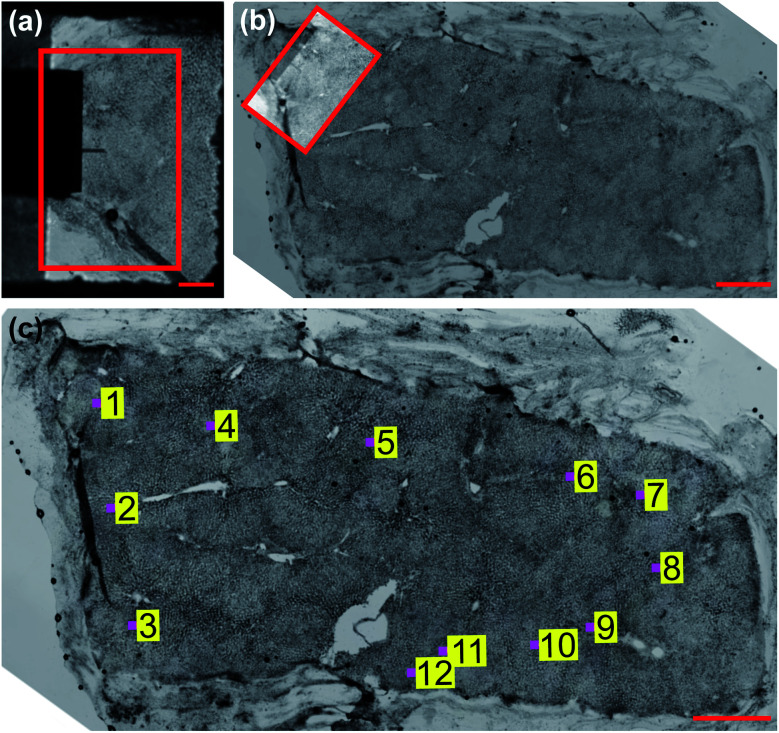
Coarse registration procedure. (a) AFM FOV at 4× magnification with region of interest (ROI) selected for registration outlined in solid red box. Scale bar 500 μm. (b) Whole-sample image of unstained liver tissue at 4× magnification with ROI from (a) overlaid. Scale bar 1 mm. (c) Coarsely localized AFM measurement sites (pink boxes, not to scale).

In this work we measured tissue sections of intermediate thickness (40 μm), which balanced image contrast with macroscopic structure (Fig. 2c), as the thin 5 μm sections typically used for histopathological assessment lack the three-dimensional ECM necessary for bulk tissue properties to be measurable. We also imaged the sections with a high dynamic range sCMOS camera rather than the camera supplied with the AFM system. The increased contrast allows for local features to be identified and AFM measurement sites to be coarsely localised using normalized cross-correlation, as illustrated in Fig. 3. Our previous work used this coarse localisation procedure to estimate the sample-wide EM by extrapolating measured values based on structural information extracted from post hoc stained images,^36^ similar to Calò *et al.*^37^ Both methods require well-aligned images of the section before and after staining, which is often not possible if the tissue has warped or been damaged during the process. In this work we eliminate the need for staining and *ad hoc* AFM measurements entirely: localisation of measured areas has been fine-tuned to obtain precisely registered pairs of unstained tissue images and EM maps, and these pairs were used to train a style transfer deep learning architecture to infer the sample-wide EM of tissue sections from low-resolution grayscale microscopy images. The resulting EM distributions contain the nanomechanical signatures of the underlying tissue pathology identified previously,^27,33–35^ and unsupervised clustering of distribution parameters provides an accurate prediction of tissue pathology, validated through pathologist assessment of *post hoc* stained sections. The use of a well-optimized prediction algorithm allows for much more rapid diagnoses compared to time-intensive diagnostic methods such as IFS analysis or *ad hoc* AFM measurements.

## Experimental design

### Sample preparation and measurement

AFM measurements were made on a total of 28 tissue samples collected from 7 patients diagnosed with colorectal or pancreatic cancer metastasis of the liver undergoing curative resection surgery, obtained through the Tissue Access for Patient Benefit program and the Royal Free Hospital.‡‡Human Tissue Authority License #11016. Ethical approval was obtained prior to all sample measurements. Patient clinical metadata is provided in Table S1 in ESI. Punches were taken from nominally healthy and cancerous regions for comparison and flash-frozen with liquid nitrogen, embedded in Tissue-Tek optimal cutting temperature (OCT) compound and cut using a cryostat (Leica CM1860) at a thickness of 40 μm. Cut sections were stored at −20 °C and thawed immediately prior to measurement. Samples were washed twice carefully with distilled water to remove excess OCT compound and immersed in a physiological buffer (Gibco Dulbecco's Modified Eagle Medium, Thermo Fisher Scientific). Excess buffer was removed and tissue glue applied using a fine glass capillary tube around the edges of the section to adhere it to the microscope slide. Once the glue had hardened tissue sections were fully immersed in 4 mL of fresh buffer held in place by a custom 3D-printed plastic frame fixed to the microscope slide with silicone paste (Fig. 1 and 2a).

A JPK NanoWizard 3 atomic force microscope (Bruker Nano GmbH) with a soft tipless cantilever and 10 μm diameter borosilicate bead with spring constant *k* = 0.08 N m^−1^ (CP-μMasch CSC12, sQube) was used to acquire on average 10–20 force maps per sample; each force map consisted of 64 force-indentation curves captured over a 10 μm × 10 μm measurement area in an 8 × 8 grid (corresponding to 1.25 μm lateral spacing). Measurement sites were selected to characterise as much of the section as possible while avoiding regions where the tissue was not well adhered to the slide or where the structure was not conducive to reliable force-indentation measurements, *e.g.* large voids or disintegrated tissue. Measurement settings used were 3.0 μm maximum indentation depth, 1.0 μm s^−1^ indentation speed and 2–5 nN of indentation force applied in force mapping mode; parameters were chosen to avoid inelastic deformation of the tissue and to allow the tissue to recover its shape after retraction. If a force-indentation curve could not be captured, *e.g.* because the tissue height was beyond the retraction range of the cantilever, the force map at that location was recorded as NaN. The cantilever sensitivity and spring constant were calibrated daily prior to each sample measurement using the JPK calibration tool's thermal noise method based on the procedure described by Hutter and Bechhoefer.^38^ The resulting force curves were analyzed using JPK analysis software to calculate bulk tissue EM by fitting the Hertz/Sneddon model for contact mechanics, taking the indenter shape into account.^39,40^

### Generating a paired image-EM map training set

**Fig. 4:**
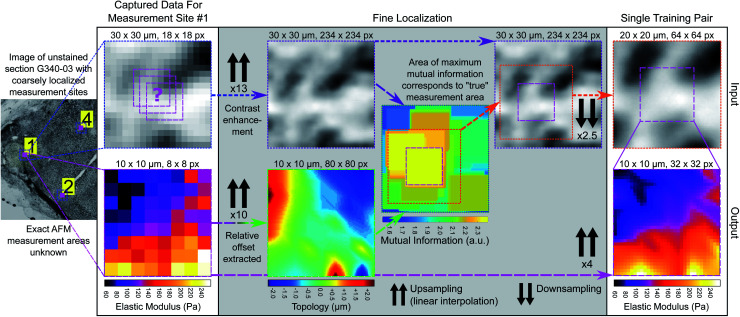
Fine registration process for generating training pairs. Blue dotted boxes outline coarsely registered 30 μm × 30 μm microscopy image patches for each measurement area at original resolution (1.625 μm pixel size). Pink dashed boxes outline 10 μm × 10 μm EM maps (input resolution 1.25 μm, output resolution 0.3125 μm) and green dashed box outlines 10 μm × 10 μm tissue topology map (intermediate resolution 0.125 μm). Purple dashed boxes outline contrast enhanced and upsampled 30 μm × 30 μm microscopy image patch (intermediate resolution 0.125 μm). Combined green/purple dashed box outlines mutual information map. Orange dotted box outlines finely registered 20 μm × 20 μm microscopy image patch (output resolution 0.3125 μm).

To learn the relationship between unstained image intensity and EM, we acquired a training set of paired microscopy images and EM maps through accurate *post hoc* spatial localisation of where the AFM measurements had been made on the bulk tissue section. This was done in a two-step registration process: the first involved coarsely registering images of the measurement site (captured with the AFM cantilever in view) on a whole-sample image of the tissue section using normalized cross correlation (see Fig. 3), followed by a finer registration procedure that used an estimate of the tissue topology to localize the measurement area at a much higher spatial resolution (see Fig. 4).

#### (A) Coarse measurement site localisation

A tiled image of each unstained tissue sample immersed in buffer (Fig. 3b and c) was acquired using an upright microscope (Olympus BX63) with a motorized sample positioning stage (Prior Scientific) and a PIFOC objective focusing stage (Physik Instrumente) prior to all AFM measurements. Images were captured at 4× magnification (Olympus PLSAPO4X, 0.16 NA) using a colour digital camera (PCO edge 5.5c) with a pixel size of 6.5 μm and sensor size of 2560 × 2160 pixels, giving a field of view (FOV) of 4.1 × 3.5 mm in size. The microscope was controlled with custom software written in LabVIEW (National Instruments) and images were stitched into a whole-sample image using an ImageJ plugin.^41^ The AFM system was mounted on an inverted optical microscope (Olympus IX73) also fitted with a colour digital camera (PCO edge 3.1c) having a smaller sensor size and FOV (3.3 × 2.5 mm), and a single image of the tissue section was captured at 4× magnification (Olympus PLSAPO4X, 0.16 NA) immediately prior to each measurement (Fig. 3a). The measurement area is occluded by the cantilever in these images hence the need for registration on the whole-sample image to extract pixel intensities for each location. A 40× magnification template image of the AFM cantilever with the tissue contact point in focus (the borosilicate bead) was captured prior to all measurements, where the measurement area could be accurately determined. To match the magnification of the unstained tissue section images (4×), a series of template images were taken of the cantilever in focus at increasing magnification and sequentially registered to create a 4× template image with a known measurement area. The approximate location of the cantilever tip could then be determined for each measurement site by calculating the normalized cross correlation between the template image and each AFM image, finding the location of the maximum correlation and using this calculated offset to determine the measurement area's location in the FOV (see Fig. S1 in ESI†). Normalized cross correlation was again used to register the AFM image with known measurement area on the whole-sample image and coarsely localise the measurement area on the sample (see Note S1 in ESI†).

#### (B) Fine measurement area localisation

A larger image patch was extracted around the coarsely-determined measurement site (approximately 30 μm × 30 μm to account for any errors during registration) and the measurement site was finely localised within that extracted area. The extracted image patch was first contrast enhanced using contrast-limited adaptive histogram equalization (CLAHE)^42^ with a 32 pixel window. The difference in AFM measurement grid spacing (1.25 μm) and camera pixel size (1.625 μm at 4× magnification) required upscaling and linearly interpolating to match the effective pixel sizes. The ratio between them was 13/10, thus the image patches were upscaled to an effective matched pixel size of 0.125 μm and the 100 μm^2^ 8 × 8 measurement area when similarly upscaled corresponded to an 80 × 80 pixel area. Additional structural information obtained during the AFM measurement was used to assist in finely localising the measurement area, specifically the tissue topology, which was estimated from the relative axial offset of the tissue contact point of the cantilever prior to each individual force-displacement curve measurement; the tissue topology map is identical in size and resolution to the EM map. Given that the direct relationship between topology and image intensity is unknown, a more general similarity metric was used for registration. The mutual information of two variables quantifies the amount of information gained about one of the variables (image intensity) from observing the other (tissue topology).^43^ It was calculated in a similar manner to 2D cross correlation, *i.e.* as a function of the displacement of the tissue topology map relative to the tissue image patch, and output as a similarity map with the same dimensions as the image patch. The 80 × 80 region of the patch corresponding to the maximum computed mutual information was taken as the finely localised AFM measurement area. The resulting image patches and corresponding EM maps were downsampled to 64 × 64 and 32 × 32 in size respectively and saved as training pairs. The training and validation set consisted of 386 image-EM map pairs in an 80/20 split (see Note S2 in ESI†).

### Style transfer network architecture and training

The style transfer architecture for converting image intensity to EM on a pixelwise basis was trained in a generative adversarial network (GAN) configuration based on an implementation of Pix2Pix^44^ using the Keras machine learning API.^45^ In the GAN configuration, a generator network produces a synthetic output image (an EM map in this case) conditioned on an input image (a microscopy image of unstained tissue) and is trained simultaneously with a discriminator network that is optimized to distinguish synthetic EM maps from real measured ones, improving the accuracy of generated or predicted EM maps. The generator consisted of a U-Net architecture^46^ with one additional downsampling layer relative to the return arm, which takes input image patches that are larger than the output EM maps (400 μm^2^*vs.* 100 μm^2^), increasing the information available to the network. This prediction architecture was also chosen to accommodate the large tissue contact area of the cantilever bead (see Fig. S2 in ESI†). The generator U-Net contained 5 downsampling blocks, each consisting of a 2D convolutional layer, a leaky ReLU layer with an alpha value of 0.2 and batch normalization (momentum 0.8), with the exception of the initial block. The return path contained 4 upsampling blocks, each consisting of a transposed deconvolution layer followed by dropout and batch normalization layers as well as a concatenation skip input layer. Image patches and EM maps were scaled to a range of ±1 allowing for a tanh activation function in the final convolutional layer, with EM values clamped between 0 and 2 kPa in accordance with expected values published in the literature (see Discussion). The discriminator consisted of a 2D average pooling layer to reduce the size of the input patch to 32 × 32, followed by four blocks, each with a 2D convolutional layer, a leaky ReLU layer with an alpha value of 0.2, batch normalization (momentum 0.8) with the exception of the initial layer and a dropout layer with a rate of 0.25. The validity was computed by a 2D convolutional activation layer. The combined generator and discriminator model was compiled with mean squared error and mean average error as loss functions with weights of 1 and 100 for the discriminator and generator respectively, and with the Adam optimizer (learning rate 0.0002, beta 0.5). The model was trained with a batch size of 16 for 10 000 epochs in Keras using a Tensorflow backend, and training took just over 3 hours on a NVIDIA Tesla V100 GPU with 32 GB of onboard memory.

### EM prediction and sample validation

**Fig. 5:**
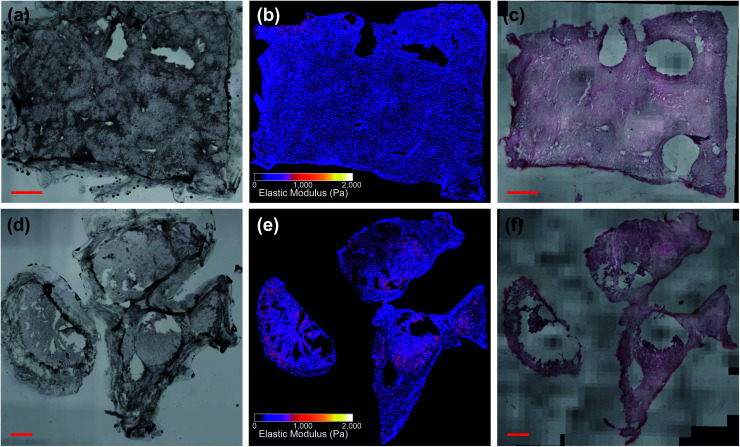
Liver tissue samples (a)–(c) G350-01 and (d)–(f) G350-02-02 (scale bars: 1 mm), with G350-01 classified as “necrotic/no tumour” and G350-02-02 classified as “tumour”. (b) and (e) Show predicted EM values of unstained images (a) and (d). (c) and (f) Show *post hoc* stained sections.

For prediction, the unstained whole-sample images were upscaled to match the effective pixel size of the training patches and CLAHE contrast enhancement was applied with a 32 pixel window size. The mismatch in input and output patch size required extracting overlapping patches from the input image using the extract_image_patches function in Tensorflow to generate a contiguous whole-sample EM prediction (see Fig. S3 in ESI†). Prediction maps were masked to ignore background pixels, fluid bubbles, tissue glue and areas where the tissue was out of focus, detached or folded (see Fig. S4 in ESI†). Fig. 5a and d show whole-sample images of unstained tissue input to the network with output EM maps shown in Fig. 5b and e. Each sample was nominally classified as “colorectal/pancreatic cancer metastasis” or “non-disease associated tissue” based on where the sample was excised from during surgery, which provided the only validation in some cases where post-measurement staining was not possible due to poor sample quality or COVID-19 restrictions. In most cases however (>70%), samples were fixed in 4% paraformaldehyde immediately after AFM measurement, stained with H&E and imaged at 20× magnification (Olympus PLN20×, 0.4 NA) in three dimensions to account for sample thickness. Volumetric stacks were converted into extended depth of field two-dimensional images and stitched together using ImageJ plugins.^41,47^ Stained whole-sample images (Fig. 5c and f) were randomly numbered and assessed by two independent pathologists who had been informed of the tissue type (liver) and the type of cancer that might be present (colorectal or pancreatic cancer metastasis). Assessment consisted of determining whether any tumour cells were present in the section and providing any other relevant information on the tissue pathology. Both pathologists classified all samples as “necrotic”, “fibrotic”, “no tumour”, “tumour”, or some combination of these four main tissue types. In one case, the nominal assessment of colorectal cancer metastasis did not match either pathologist's assessment of “no tumour”; pathologist assessments were always used as the final validation label (see Fig. S5 in ESI. Full details for all samples are also given in Table S2 in ESI.†). We note that stained histopathology images were not used during the registration procedure or while training the prediction algorithms; they were only used for validation of tissue pathology diagnoses. As a final validation test, histograms of the measured EM values were compared with both the sample-wide predicted values and the predicted values of the localized measurement areas only (see Fig. S6 in ESI†).

### Predictive clustering

**Fig. 6:**
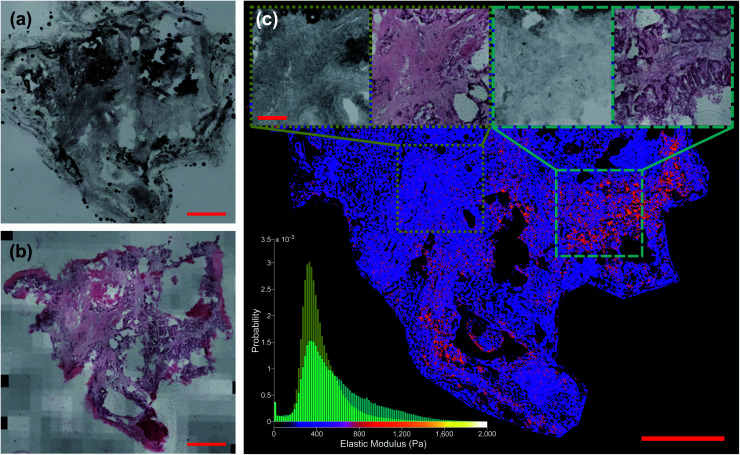
Liver tissue sample G340-03-02 classified as “tumour”. (a) Shows unstained section and (b) shows *post hoc* stained section (scale bars 1 mm). (c) Shows network prediction of EM values with (a) as input. Insets: unstained and stained ROIs (scale bar 250 μm). Histogram in bottom left shows distribution of predicted EM values for each ROI. *n* = 350, 166 for narrower distribution in yellow and *n* = 313, 293 for broader distribution in cyan.

**Fig. 7:**
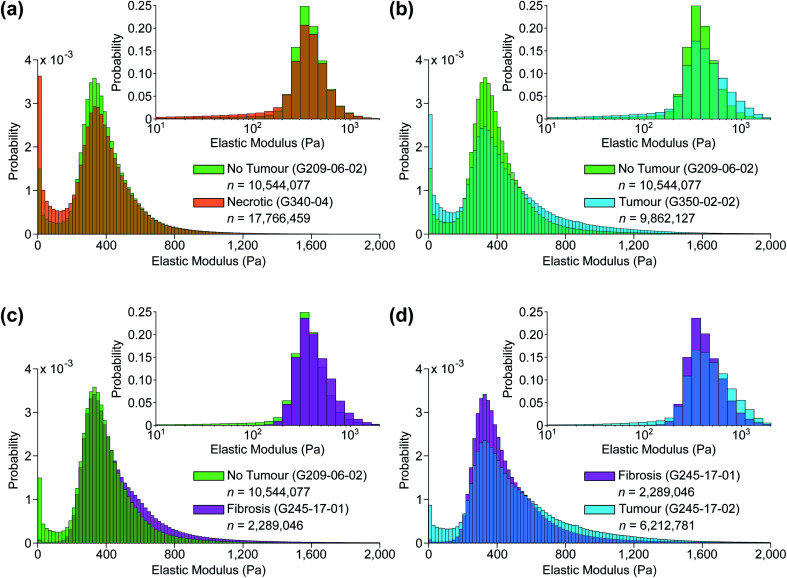
Typical predicted EM distributions of four tissue types present in the sample set. Pairwise comparisons between (a) non-tumour and necrotic, (b) non-tumour and tumour, (c) non-tumour and fibrotic, and (d) fibrotic and tumour tissue sections. Insets: same distributions plotted using a base 10 logarithmic scale.

**Fig. 8:**
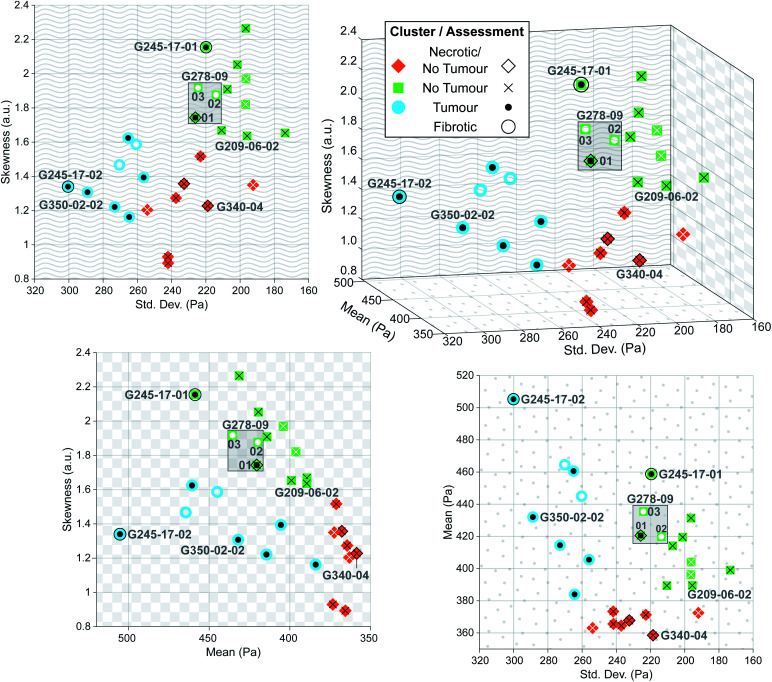
Three-dimensional plot of parameters extracted from each predicted sample distribution, with solid colour shape indicating predicted cluster, and overlaid symbol indicating clinical assessment. Black symbols denote confirmed assessments of *post hoc* stained sections, whereas white symbols denote nominal assessments for samples that could not be stained. Two-dimensional projections onto each axis are shown to illustrate direct relationships between each of the extracted parameters.

Fig. 6c shows the distribution of inferred EM values of two ROIs of a typical sample, and the tissue structure visible in the H&E image has a clear influence on the shape of these distributions. Fig. 7 also shows typical sample-wide distributions for several different tissue types present in the sample set. The following parameters were calculated for each inferred whole-sample distribution to characterize its shape, and quantify how it changes due to the tissue structure that is revealed through staining: the mean, which estimates the overall stiffness of the tissue, the standard deviation, which indicates heterogeneity of values, and skewness, a measure of the “tailedness” of a distribution or the presence of outliers.^48^ The extracted parameters were then used to group the samples into predictive clusters in an unsupervised manner using a Gaussian mixture model. Unsupervised clustering refers to the fact that no labels were assigned to the distributions during clustering, and the algorithm identified patterns in the extracted parameters and grouped the sample distributions accordingly; the assigned labels were then compared to the actual validation labels obtained from the nominal or histopathological assessments. The optimal number of clusters (3) was determined without *a priori* knowledge of any sample pathology using the evalclusters MATLAB function (MathWorks) according to the gap criterion^49^ with a squared Euclidean distance metric. A 3-component Gaussian mixture model was fit to the extracted parameter dataset using the fitgmdist function with diagonal covariance matrices, a probability tolerance of 1 × 10^−6^, a regularization value of 0.01 and 10 replicates. Starting values were chosen at random with uniform mixing proportions. The resulting mixture model was used to cluster the samples *via* the cluster function, and the assigned cluster for each sample served as a prediction of tissue pathology based on the most frequent nominal or histopathological assessment found in that cluster (see Fig. 8).

## Results and discussion

Fig. 5 shows two samples taken from patient G350: the non-disease associated tissue sample G350-01 shown in the top row was assessed as “mostly necrotic/no tumour”, and the colorectal cancer metastasis sample G350-02-02 shown in the bottom row was confirmed to have “tumour [cells] present throughout the section”, identified as metastatic adenocarcinoma. Fig. 6 shows a sample excised from patient G340 with two ROIs shown in more detail, including histograms of the predicted EM values for each of these regions. This sample (G340-03-02) was also confirmed to have metastatic adenocarcinoma present. While the heterogeneity of the inferred EM may be visually apparent in the tumour samples, it is the distribution of values that contains relevant, quantifiable signatures of the underlying tissue pathology, as shown in the inset histograms in Fig. 6c.

Fig. 7 shows histograms of the sample-wide predicted EM maps for four typical samples, illustrating how tissue structure affects the distribution shape. The magnitude and range of EM values agree with previous characterizations of liver tissue as summarized by Stylianou *et al.*: measured EM profiles of healthy liver tissue are generally unimodal in shape with means of up to 1 kPa and widths in the hundreds of pascals.^32^Fig. 7a shows necrotic tissue compared to normal liver, where the lack of fibrous proteins that contribute to bulk stiffness results in a lower overall EM, in agreement with previously published work.^32^ Fibrotic tissue has a much larger EM in general, again typified by a unimodal distribution of EM values with a larger mean than healthy tissue,^50^ and this increased EM is clear in Fig. 7c.

The predicted whole-sample EM distributions shown in Fig. 6c and 7 differ in shape from those observed in previous works however, where thick (≥1 mm) tissue sections were measured with AFM cantilever tips tens of nm in size, resolving individual cells and collagen fibres.^27,32,33^ The AFM measurements in this work were made using a tipless cantilever and 10 μm diameter borosilicate spherical bead on 40 μm thick sections, as finer tips were found to puncture the sections, generating spurious measurements. Previous high-resolution measurements of liver tissue with metastatic cancer present resulted in starkly bimodal EM profiles, characterized by lower and higher elasticity peaks (LEPs/HEPs) corresponding to highly motile cancerous cells and the stiffening ECM.^32^ Tian *et al.* measured a liver sample with colon cancer metastasis and extracted LEP/HEP values of 0.65 ± 0.13 kPa and 2.26 ± 0.60 kPa,^51^ and the LEP is in agreement with published EM values of high motility colon cancer cells (0.4–0.6 Pa).^52,53^ This bimodality is clearly not present in the tumour tissue distribution shown in Fig. 7b due to the reduced spatial resolution of the AFM scanning, and recent work by Calò *et al.* characterizing human liver tissue using 5 μm cantilevers also measured EM distributions similar in shape and magnitude to those presented here.^37^

Despite this reduction in spatial resolution, the biomechanical structure that gives rise to the bimodality resolved with a finer tip is still discernible from the shape of the tumour distribution compared with normal liver shown in Fig. 7b, and the extracted parameters allow for accurate classification of tissue pathology through unsupervised clustering. Predictions are shown in the three-dimensional plot in Fig. 8 where solid coloured shapes indicate the cluster and overlaid symbol(s) denote pathologist or nominal assessment. Several samples resulted in discordant diagnoses, denoted by overlapping symbols. Black symbols show pathologist assessments and white symbols denote nominal assessments where staining could not be completed. The two clusters represented by blue circles and green squares were assigned “tumour” and “no tumour” respectively, whereas the third cluster represented by orange diamonds was assigned the label “necrotic/no tumour” to distinguish it from the first “no tumour” cluster as it contained two samples that had been confidently assessed by one of the pathologists as mostly necrotic. It is more likely however that this is simply a second group of non-tumour samples. The predictive clustering achieved an overall classification accuracy of 89.2% (*n* = 28), where a prediction was correct if the name of the predicted cluster matched at least one part of the pathological assessment. The accuracy for fully validated samples was 95% (*n* = 20) (full details for all samples are given in Table S2 in ESI†), which is comparable to the reported accuracy of 95% for IFS analysis in the diagnosis of liver lesions when compared with gold-standard FFPE histopathology.^54^

Automated image classification algorithms generally rely on feature extraction using sequential convolutional layers, and the feature representation is optimized to achieve the highest classification accuracy. In histopathology, samples are typically imaged in colour at high resolution, resulting in a very large feature space. Rather than training a network to make a pathological diagnosis from these features, we instead trained a style transfer network to convert grayscale image intensity to EM on a pixelwise basis. The advantage of generating these sample-wide EM maps based on biomechanical measurements rather than mimicking an H&E stain or replicating a histopathological diagnosis entirely is the objectivity of the parameters that can be extracted to identify the tissue pathology, decoupling margin estimation from the inherently subjective process of visual image-based assessment. IFS analysis in particular is vulnerable to interobserver variability due to freezing artefacts and the consequent reduction in image quality.^20–23^ AFM measurements characterising tissue are vulnerable to significant variabilities in the form of random and systematic errors as outlined by Schillers *et al.*,^55^ and are particularly sensitive to errors from calibration of the cantilever; the method presented here uses multiple measurements of different tissue samples carried out across a significant period of time (approximately 15 months) to train a generalized algorithm, allowing for the effects of random error to be averaged out. Our method also relies on inferring relative differences in the tissue sections and uses these differences to make an informed diagnosis rather than the absolute value of EM measurements, which reduces the impact of systematic error inherent to many AFM measurement procedures.

The sensitivity of the classification algorithm is most starkly demonstrated by samples G245-17-01 and 02 shown in Fig. 7d and 8; both were similarly classified by one pathologist as fibrotic with adenocarcinoma likely present on the right-hand side. The network inference of G245-17-01 was restricted to the left portion of the sample, as the area containing the potentially cancerous tissue was out of focus, which tended to generate spurious EM predictions (see Fig. S4 in ESI†). This sample was misidentified by the clustering algorithm as “no tumour”; fibrotic samples were not isolated into a fourth cluster, likely because this was the only sample that was truly fibrotic with no cancer present in the prediction area. G245-17-02 in contrast was correctly classified as “tumour”, agreeing with the same pathologist's assessment of likely adenocarcinoma in the lower right corner, and the EM prediction was made on the entire section. Mitigating the effects of out of focus regions would significantly improve the accuracy of our method (see next section for further discussion). The prediction and clustering algorithm also correctly classified all sections extracted from patient G209 as “no tumour” despite an incorrect nominal assessment; one of the punches (G209-05) was nominally classified as pancreatic cancer metastasis during resection, however none of the three sections from this punch contained any cancerous tissue, as confirmed by both pathologists' assessment (see Fig. S5 in ESI†).

Sample G278-09-01 was assigned a nominal classification of “tumour” during excision, however the histopathological diagnosis was discordant. One assessment queried the presence of necrosis and possible adenocarcinoma, while the other classified the section as normal liver, with both accompanied by a strong caveat that classification was difficult due to the poor quality of the stained section (The thickness of the samples contributed to difficulties in validation as standard histopathology samples are usually 5 μm thick rather than 40 μm). This sample was labelled “necrosis/tumour/no tumour”, and while this partially matched the predicted cluster of “no tumour”, this prediction was considered to be only weakly validated. Additional sections from this punch (G278-09-02 and 03) were also of poor quality and could not be stained as they dissolved during post-measurement fixation. Their cluster prediction of “no tumour” did not match the nominal label, but based on the diagnosis of G278-09-01, we suspect that the prediction may have been correct; without a histopathological assessment to confirm this however, these sections were considered to be incorrectly classified. Improving the validation process would be a priority of future work on this technique.

Finally, we acknowledge the sample size and computational complexity of our prediction algorithm as the limitations of this proof-of-concept study. Sample measurement was curtailed due to the COVID-19 pandemic, and samples that had been obtained from the biobank could not be measured prior to the national lockdown in the United Kingdom in March 2020 and are no longer viable. All data from sample G209-06-03 was excluded from the training set and served as a validation sample, and was correctly classified by the algorithm. Enlarging the sample size to provide additional training data as well as additional validation samples that have not been previously measured would form a major component of future work. The major advantage of an automated prediction and classification architecture is the potential for virtually instantaneous diagnoses, provided sufficient computational resources are available. Our existing algorithm is not instantaneous however, and the processing time is comparable to the lower bound of typical IFS analyses (15 minutes). Our algorithm is not optimized or parallelized, and this would also be a focus of additional development on this method (a description of the computational bottlenecks in our prediction algorithm is provided in the description of Fig. S3 in ESI†).

## Conclusions

The workflow presented here spatially localised AFM measurements of tissue with unprecedented accuracy, producing a high-quality training set for a style-transfer architecture to infer biomechanical information from low-magnification images of unstained liver tissue. To our knowledge, it is the first publicly available dataset of its kind. Fig. 6c shows in particular how the network is able to predict regions of high EM that correspond to increased levels of the fibrous ECM protein collagen as evident in the stained image, despite an absence of obvious features in the unstained image. The network was also able to infer regions of lower EM due to the influence of highly motile tumour cells, albeit at a reduced spatial resolution than in previous studies. The influence of the structure that gives rise to this heterogeneity is still apparent in the predicted sample-wide distributions, and extracted parameters quantifying the shape of these distributions were clustered to classify the underlying tissue pathology with comparable accuracy to standard IFS.^54^ The prediction architecture presented here has the potential to bypass the time-consuming staining and assessment procedure that has limited the use of IFS in certain procedures despite its significant clinical benefits. It also replaces the subjective assessment of high-resolution colour images with objective analysis of biomechanical data, extracting signatures of cancerous progression in tissue that are well supported in the literature. The required imaging modality (brightfield microscopy) is fast, label-free, inexpensive and familiar to clinicians.

While our results agree with previously reported values for liver tissue,^32,37,50–53^ the network inference and clustering prediction could be fine-tuned further with measurement of additional samples. The difficulty in obtaining well-aligned and high-quality images of *post hoc* stained samples for validation is clear from the samples shown in Fig. 5d and f, and future work would incorporate the imaging methods described previously to generate pseudo-H&E stained images for more reliable confirmation of pathology, as they are well-suited to thick tissue and are non-destructive.^14–16^ Novel widefield imaging methods with large fields of view and depths of field would enable a whole-sample image to be captured in a single exposure, and increased depth of field would rectify the issue seen with one sample where some of the tissue was out of focus, affecting the accuracy of EM inference.^56–58^ Due to the network architecture requiring overlapping input patches to generate a contiguous whole-sample EM map, the prediction process is time and memory-intensive, and increasing the computational efficiency of our implementation will form a significant focus of future work. Additional imaging modalities could also be introduced during the AFM measurement workflow such as phase contrast or autofluorescence, which would provide more information to the network during training. Acquiring additional samples and enlarging the training set would only improve the quality of the EM prediction and clustering algorithm, and we expect it would generalize well to other tissue types that also exhibit these nanomechanical signatures of tumour progression.

## Data availability

All raw and processed data generated in this work are publicly available and accessible at https://www.ucl.ac.uk/interventional-surgical-sciences/afm-liver-tissue-data. For the purpose of open access, the authors have applied a CC BY public copyright licence to the dataset.

## Code availability

The MATLAB code used to register the AFM measurement sites and extract the training patches for the GAN is publicly available at https://github.com/lydiazajiczek/AFM-Registration. A pseudocode description of the registration and training patch extraction is provided in Notes S1 and S2 in ESI.† The Python code for training the GAN and predicting EM from unstained input images is publicly available at https://github.com/lydiazajiczek/AFM_GAN. Both repositories are licensed under an MIT open source license.

## Author contributions

L. N. Z. and C. E. conceived the study, designed the experiments, collected the data and wrote the paper; they were assisted by N. C. with the study design. A. R. and S. B. advised on training the network. M. J. and L. H. provided pathological assessment of the stained tissue samples. E. M. assisted with cutting the sections and staining them after measurement. A. G. and B. D. provided tissue samples. V. P., D. F- R., M. S. and D. S. reviewed and edited the manuscript.

## Conflicts of interest

There are no conflicts to declare.

## Supplementary Material

NA-003-D1NA00527H-s001
