# Supplementary material for: Stain-free identification of tissue pathology using a generative adversarial network to infer nanomechanical signatures
Source: Nanoscale Adv. 2021 Sep 2;3(22):6403–14. doi: 10.1039/d1na00527h (PMC8577366; doi:10.1039/d1na00527h)
Supplement: NA-003-D1NA00527H-s001 [file NA-003-D1NA00527H-s001.pdf]

Electronic Supplementary Material (ESI) for Nanoscale Advances.
This journal is © The Royal Society of Chemistry 2021

Supplementary Information

Table S1 Summary of all patients: clinical metadata.

Patient ID	Gender	Year of Birth	Provisional Diagnosis	Procedure Type	Date of Surgery	Age Cohort	Sample Received
G159	Female	1957	Colorectal cancer metastasis	Left hepatectomy	02/03/2020	60-69	10/03/2020
G209	Female	1960	Pancreatic cancer metastasis	Right hepatectomy	12/07/2018	50-59	11/12/2018
G245	Male	1990	Colorectal cancer metastasis	Right hepatectomy	22/08/2018	20-29	21/09/2018
G278	Female	1966	Colorectal cancer metastasis	Right hepatectomy	01/10/2018	50-59	11/12/2018
G340	Female	1954	Colorectal cancer metastasis	Left hepatectomy	13/11/2019	60-69	16/12/2019
G350	Female	1965	Colorectal cancer metastasis	Wedge liver resection	22/11/2019	50-59	27/11/2019
G3113	Male	1940	Colorectal cancer metastasis	Left hepatectomy	23/10/2017	70-79	16/12/2019

Coarse Measurement Area Registration Using Cantilever Template

The procedure for coarsely localising the AFM measurement area for a single site is shown in Fig. S1. A 40× magnification template image of the cantilever with the bead (tissue contact point) in focus was captured prior to all measurements, where the 10 μm^2 measurement area could be accurately determined (Fig. S1a). A series of template images were taken of the cantilever assembly in focus at 40×, 10× and 4× magnifications and registered to localise the measurement area on a 4× magnification template image (Fig. S1b) to match the magnification of AFM FOV images (Fig. S1d). The location of the cantilever tip was then determined for each measurement site by calculating the normalized cross correlation between the cropped and thresholded template image (Fig. S1c) and the AFM FOV (Fig. S1d) and using the calculated offset to localise the measurement area on the AFM FOV (Fig. S1e). Each FOV with known measurement area is then registered on the whole sample image as shown in Fig. 3 in the main text.

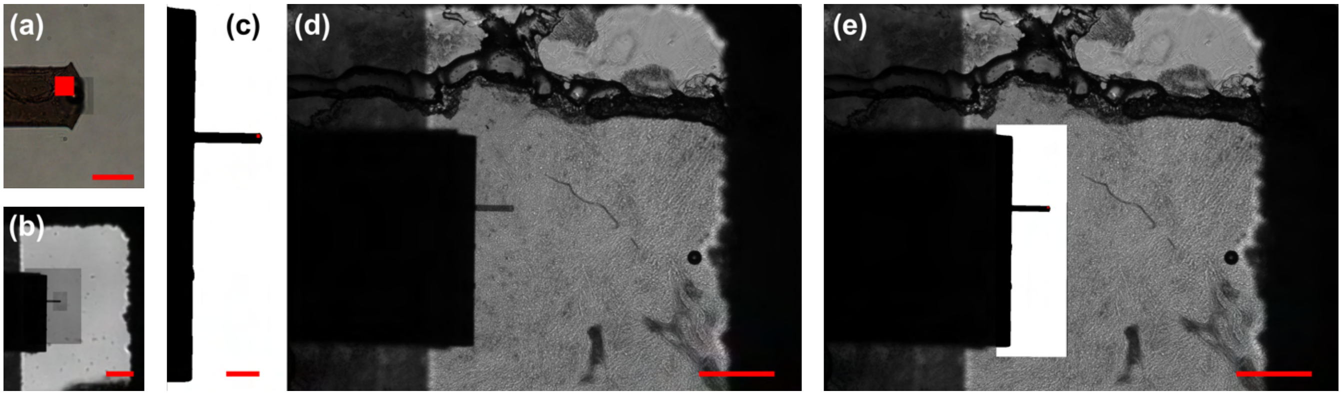

Fig. S1 Coarse registration procedure for each individual AFM measurement site. (a) 40× image of cantilever tip with bead in focus where highest intensity pixel corresponds to tissue contact point. 13 μm^2 measurement area shown in red. Scale bar 25 µm. (b) Composite of 40×, 10× and 4× images of cantilever in focus with coarsely localised measurement area on template 4× image of cantilever. Scale bar 500 µm. (c) Cropped and thresholded image in (b) with measurement area shown by red box (to scale). Scale bar 125 µm. (d) AFM FOV image showing occlusion of sample by AFM cantilever assembly. Scale bar 500 µm. (e) Template image (c) overlaid on (d) showing localisation of measurement area on single measurement site AFM FOV. Scale bar 500 µm.

Note S1 AFM Registration Pseudocode

Repeat following code for p of R measured samples:

Inputs

- $W_p, 1 \le p \le R$: Whole-sample grayscale microscopy image of pth liver tissue sample
- $\{A_k\}_p, 1 \le k \le N, 1 \le p \le R$: Set of N AFM FOV grayscale microscopy images corresponding to N unique measurement sites of pth tissue sample
- T: Cantilever template image
- $\{x_i, y_i\}, 1 \le i, j \le 8$: Known measurement area pixel indices of T

1. Determine rotation angle θ_c of cantilever template T relative to first AFM FOV A_1 using SURF algorithm [1]
2. Rotate $T \to T_\theta$
3. Calculate normalized cross correlation map c_T between A_1 and T_θ
4. Find offset indices of maximum correlation $x_{\text{off}}, y_{\text{off}} = \max(c_T)$
5. Pixel indices of AFM measurement area localised on AFM FOV image are equal to
   $\{x_{Ai}, y_{Aj}\} = x_{\text{off}}, y_{\text{off}} + \{x_i, y_i\}, 1 \le i, j \le 8$
   (Note: $\{x_{Ai}, y_{Aj}\}$ will be identical for all $\{A_k\}_p$)
6. Determine rotation angle θ_A of A_1 relative to whole-sample image W using SURF algorithm [1]
   (Note: θ_A will be identical for all $\{A_k\}$)
7. Repeat following code for k of N measurement sites:
   7.1. Rotate $A_k \to A_{k\theta}$
   7.2. Calculate normalized cross correlation map c_W between W and $A_{k\theta}$
   7.3. Find offset indices of maximum correlation $x_{\text{off}}, y_{\text{off}} = \max(c_W)$
   7.4. Pixel locations of kth AFM measurement area are equal to
   $\{x_{Wi}, y_{Wj}\}_k = x_{\text{off}}, y_{\text{off}} + \{x_{Ai}, y_{Aj}\}, 1 \le i, j \le 8$

Outputs

- $\left\{\{x_{Wi}, y_{Wj}\}_k\right\}_p, 1 \le i, j \le 8, 1 \le k \le N$: Set of N AFM measurement area pixel indices corresponding to N unique measurement sites of pth tissue sample

References

1 H. Bay, T. Tuytelaars and L. Van Gool, *Computer Vision – ECCV 2006*, Lecture Notes in Computer Science, Berlin, 3951st edn, 2006, pp. 404–417.

Note S2 Training Pair Extraction Pseudocode

Repeat following code for p of R measured samples:

Inputs

- $W_p, 1 \le p \le R$: Whole-sample grayscale microscopy image of pth liver tissue sample
- $\{\{x_{Wi}, y_{Wj}\}_k\}_p, 1 \le i, j \le 8, 1 \le k \le N, 1 \le p \le R$: Set of N AFM measurement area pixel indices corresponding to N unique measurement sites of pth tissue sample
- $\{E_k\}_p, 1 \le k \le N, 1 \le p \le R$: set of N elastic modulus maps E corresponding to N unique measurement sites of pth tissue sample
- $\{T_k\}_p, 1 \le k \le N, 1 \le p \le R$: set of N tissue topology maps T corresponding to N unique measurement sites of pth tissue sample

Repeat following code for k of N measurement sites:

1. Extract subimage W_k from W_p centred on $\{x_{Wi}, y_{Wj}\}_k$ plus search window size s
2. Upscale W_k from original spatial resolution of 1.625 μm to 0.125 μm using linear interpolation, apply CLAHE contrast enhancement[42] $W_k \rightarrow W_k''$
3. Upscale T_k from original spatial resolution of 1.25 μm to 0.125 μm using linear interpolation $T_k \rightarrow T_k''$
4. Calculate mutual information map m between W_k'' and T_k'', where[43]

$$m = \sum_W \sum_T p_{WT}(x,y) \log\left(\frac{p_{WT}(x,y)}{p_W(x,y)\, p_T(x,y)}\right),$$

   - $p_{WT}(x,y)$ refers to the joint probability mass function (PMF) of W and T and is equivalent to computing the joint histogram of image intensity W and tissue topology T, or more generally, the co-occurrence matrix
   - $p_W(x,y)$ and $p_T(x,y)$ are the marginal PMFs of W and T, respectively, and are equivalent to computing the normalized histograms of image intensity W and tissue topology T
   - Note: 256 bins were used for all histogram calculations as W are grayscale 8-bit images

5. Find indices $\{x_{mi}, y_{mj}\} = \max(m), 1 \le i, j \le 80$
6. Extract subimage Z_k'' from W_k'' centered on $\{x_{mi}, y_{mj}\}$, double the size of T''
7. Downsample Z_k'' to training resolution of 0.3125 μm (64×64 pixels) $Z_k'' \rightarrow Z_k'$
8. Upsample EM_k from original spatial resolution of 1.25 μm to training resolution of 0.3125 μm using linear interpolation (32×32 pixels) $EM_k \rightarrow EM_k'$
9. Save Z_k', EM_k' as kth training pair

Outputs

- $\{\{Z', EM'\}_k\}_p, 1 \le k \le N, 1 \le p \le R$: set of N training pairs of microscopy images Z'' and elastic modulus maps EM' extracted from pth tissue sample

References

42 S. M. Pizer, E. P. Amburn, J. D. Austin, R. Cromartie, A. Geselowitz, T. Greer, B. ter Haar Romeny, J. B. Zimmerman and K. Zuiderveld, *Computer Vision, Graphics, and Image Processing*, 1987, **39**, 355–368.

43 T. M. Cover and J. A. Thomas, *Elements of Information Theory*, Wiley, 2006, p. 792.

Tissue Contact Area of 10 μm Diameter Bead

Fig. S2a shows the calculation of the tissue contact area A of the 10 μm diameter bead at an indentation depth z. Figs. S2b and c compare the contact area to the size of the measurement grid for (b) an average indentation depth of 2 μm, which is 50 μm^2, and (c) at the maximum indentation depth of 3 μm (63 μm^2). While some measurement locations overlap, we found that using a fine (oversampled) measurement spacing allowed us to record a large number of force-indentation curves to better characterize each measurement area. It was not always possible to record a measurement at each point in the sampling grid due to a number of factors, including structural degradation or detached tissue, air bubbles or height differences beyond the retraction range of the cantilever. The large contact area and oversampled measurement spacing influenced the design of the prediction architecture, which used input image patches that were double the size of the output EM maps (64×64 pixels 20 μm $\times 20$ μm in size vs. 32×32 pixels 10 μm $\times 10$ μm in size).

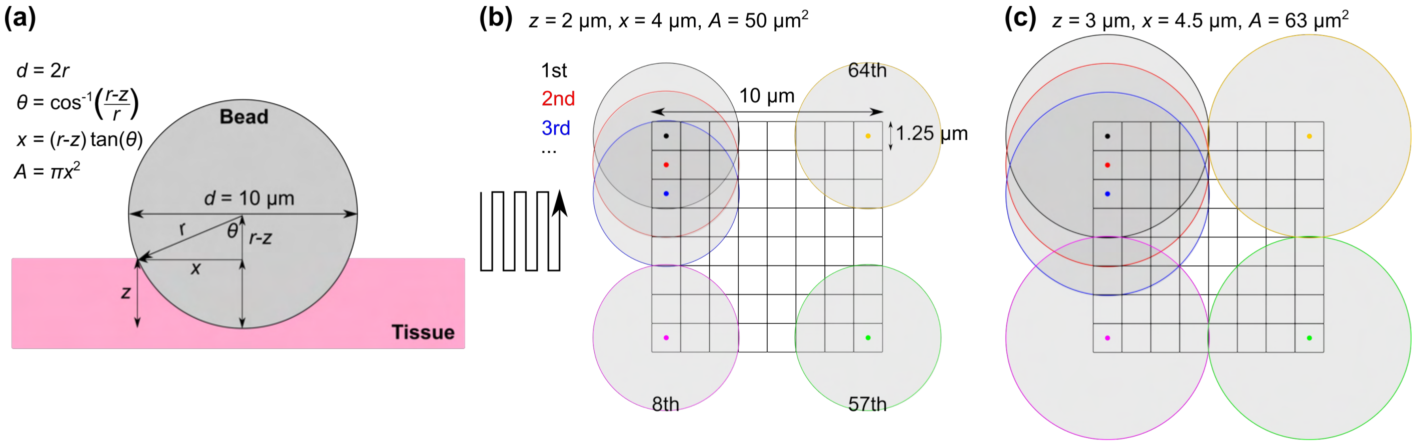

Fig. S2 (a) Calculation of circular tissue contact area of spherical bead of diameter $d = 10$ μm at indentation depth z. Comparison of tissue contact area to measurement grid for (b) average indentation depth $z = 2$ μm and (c) maximum indentation depth $z = 3$ μm.

Contiguous Prediction With Overlapping Input Patches

Fig. S3 shows an illustration of the EM prediction process using the trained GAN described in the main text. The mismatch in size of the training patches and in the structure of the generator U-Net required overlapping input patches to be extracted from the input image (using the `extract_image_patches` function in the Tensorflow library) to generate a contiguous EM prediction for the entire sample on a pixelwise basis. The input image is zero-padded at the edges to account for the mismatch in size. The output of the `extract_image_patches` function (stacks of overlapping patches) could not be passed directly to the existing Keras prediction model and thus was not parallelized, meaning the individual patches were predicted on sequentially, contributing to the significant processing time required to predict on a single input image (approximately 15 minutes on a NVIDIA Tesla V100 with 32 GB of onboard memory for a typical image 11.4 MB and 3744 x 3216 pixels in size). Future work would involve fully parallelizing and optimizing the algorithm.

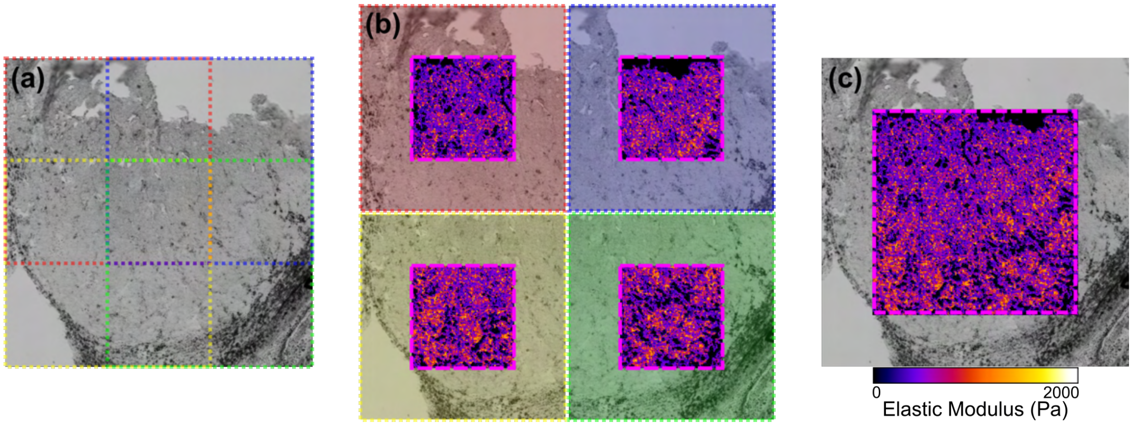

Fig. S3 Prediction process for microscopy images of unstained human liver tissue. (a) Dotted coloured boxes showing the extraction of overlapping input patches (not to scale: prediction occurs on 64×64-pixel input patches resulting in 32×32-pixel output patches). (b) Dashed pink boxes in centre of each input patch indicate output map of predicted EM values (also not to scale). (c) Contiguous prediction of EM of input image in (a).

Masking of Input Images Prior to EM Inference

Fig. S4a shows the prediction mask area for sample G245-17-01 which ignores out-of-focus material such as that on the right-hand side of the sample as such material caused the network to output spurious EM values. The post hoc stained sample shown in Fig. S4b was classified by one pathologist as fibrotic with likely adenocarcinoma on the right-hand side, where the stained tissue appears to have come away from the slide. The sample was classified as “No Tumour" as the prediction area only contains fibrotic tissue.

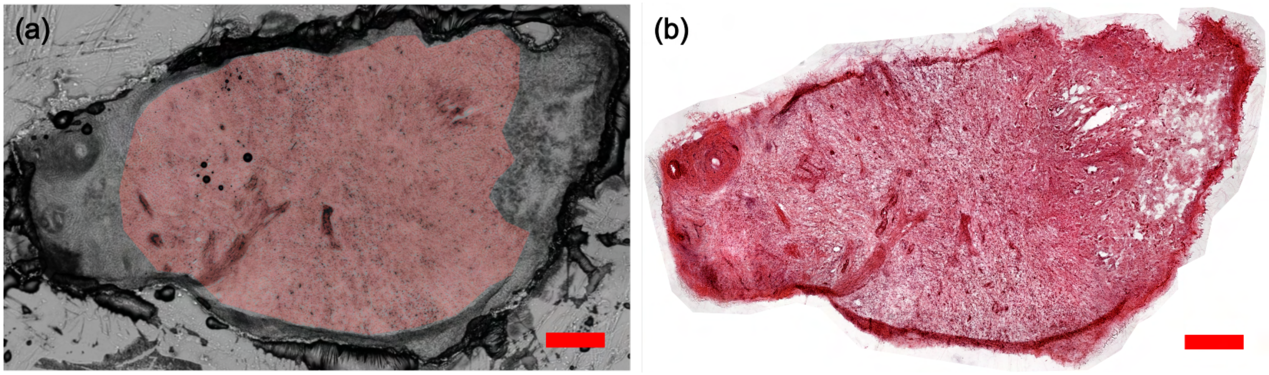

Fig. S4 Liver tissue sample G245-17-01. (a) Unstained image input to the network for EM inference with prediction mask area overlaid. Scale bar 500 µm. (b) H&E stained image post-measurement. Scale bar 500 µm.

Prediction is Robust to Incorrect Nominal Tissue Assessment

Fig. S5 shows two samples of nominally different tissue types. Sample G209-05-02 was nominally classified as “Tumour", however histopathological assessment (Figs. S5c and f) confirmed that no cancerous tissue was present in either of the sections. Sample G209-06-02 was nominally and histopathologically classified as "No tumour". The predicted distributions of elastic modulus values are almost identical for the two samples; both were correctly classified by the algorithm as "No tumour".

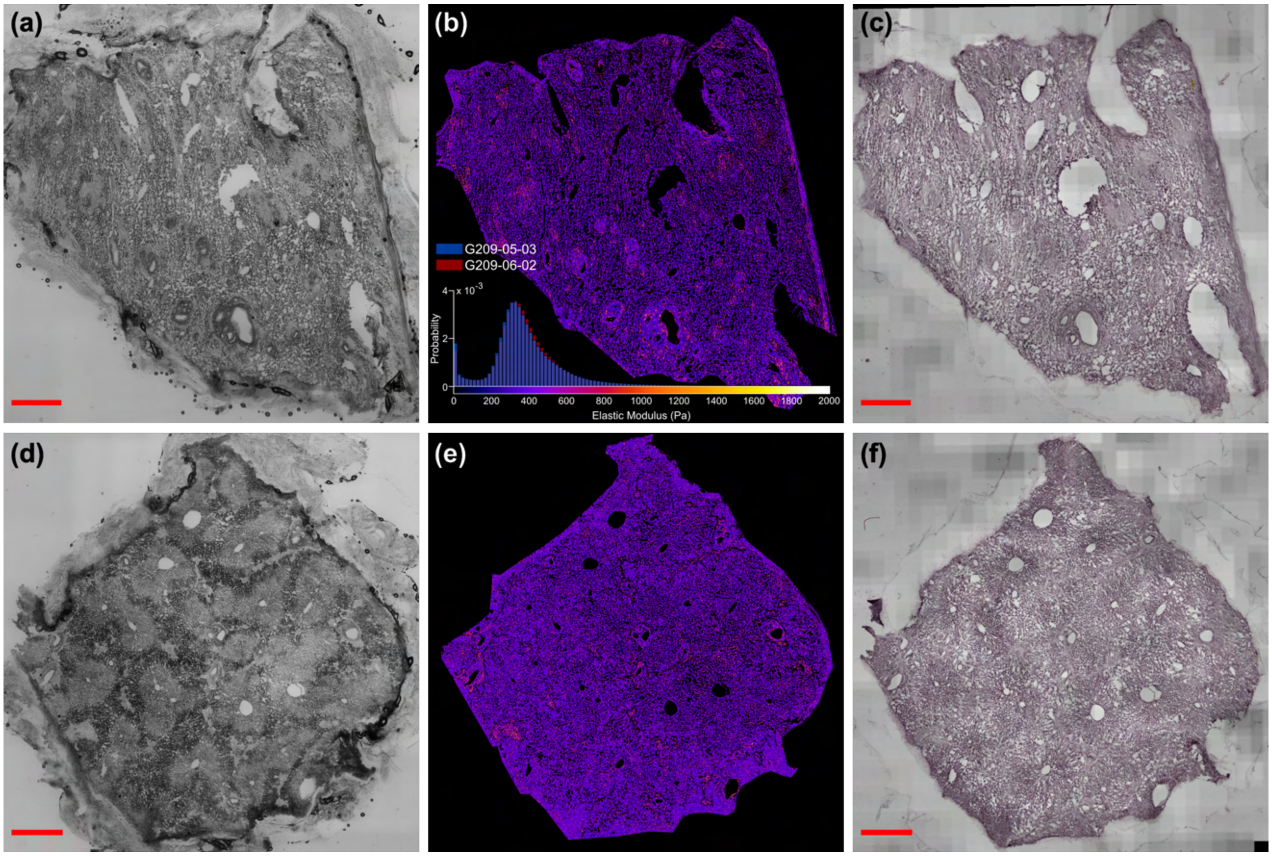

Fig. S5 Liver tissue samples (a)-(c) G209-05-02 and (d)-(f) G209-06-02. (b) and (e) show predicted elastic modulus values with unstained images (a) and (d) as input (scale bars 1 mm). Inset of (b): Overlaid histograms of predicted values in (b) and (e). (c) and (f) show stained sections used for validation (scale bars 1 mm).

Comparison of Predicted and Measured EM Values

Fig. S6 compares the measured and predicted values for sample G159-08. Fig. S6a compares the histogram of measured values ($n = 835$) with the histogram of all non-NaN pixels of the predicted whole-sample distribution ($n = 12,047,665$), hence the histograms are normalized to the relative probability of values. Fig. S6b compares the histogram of measured values ($n = 835$) with the histogram of predicted values corresponding to localized measured areas only ($n = 896$), with the histograms showing raw counts of EM values. Note that the number of datapoints does not exactly match as force-indentation curves could not always be recorded for all locations in the 64×64 measurement area. This plot illustrates that while the algorithm shows reasonable agreement with the measured values locally, overall the measured values are not representative of the sample-wide distribution of values; the predictions therefore can only be validated through diagnostic accuracy in classifying the sample pathology. Overlays identical to Fig. S6 are provided for all samples in the publicly available dataset in the subfolder "AFM_predictions", named `comparison_histogram.png`.

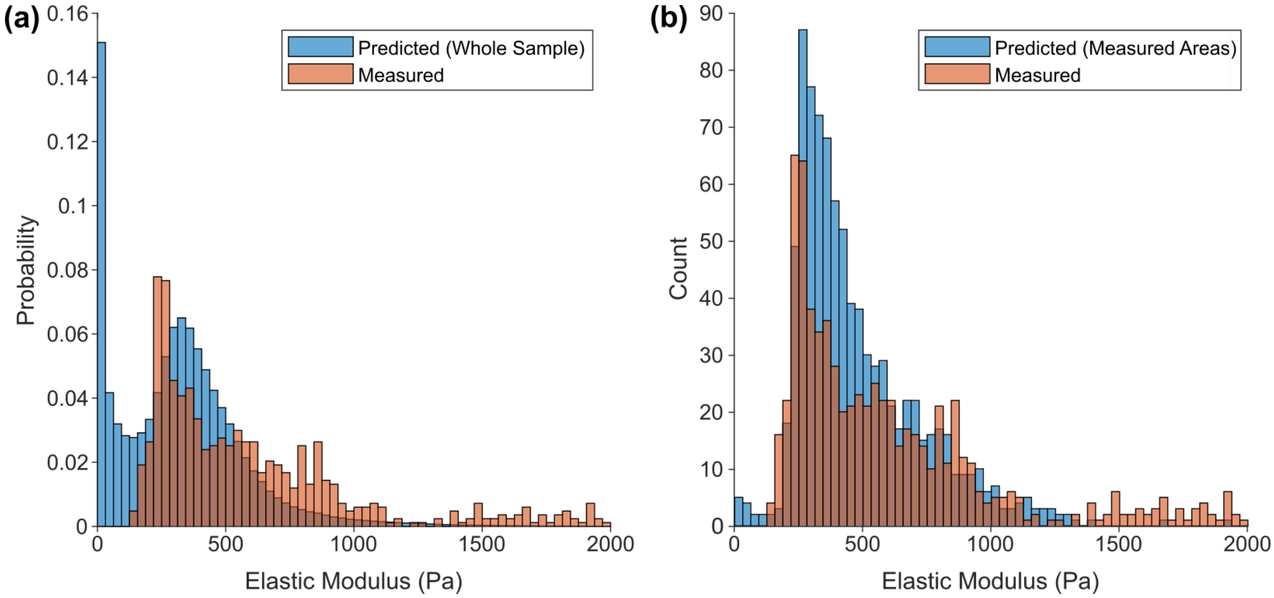

Fig. S6 Histograms of predicted and measured EM values for sample G159-08. (a) Whole-sample predicted ($n = 12,047,665$) vs. measured values ($n = 835$), normalized to relative probability of observations. (b) Predicted ($n = 896$) vs. measured values ($n = 835$) for localized measurement areas only, normalized to raw count of observations.

Table S2 Summary of all measured samples: nominal tissue type, pathologist assessment, assigned label, predicted cluster, posterior probability of sample belonging to predicted cluster, and classification score.

Sample	Nominal Assessment	Pathologist Assessment 1	Pathologist Assessment 2	Label	Cluster	p_{cluster}	Score
G159-08	Colorectal cancer metastasis			Tumour	Tumour	0.9996	✓
G159-09	Non-disease associated			No tumour	No tumour/ Necrotic	0.9647	✓
G209-05-01	Pancreatic cancer metastasis	Liver parenchyma, no tumour	Cannot confidently say what this is	No tumour/ Parenchyma	No tumour/ Necrotic	0.9989	×✓
G209-05-02	Pancreatic cancer metastasis	No tumour	Difficult to evaluate, likely no tumour	No tumour	No tumour/ Necrotic	0.9988	✓✓
G209-05-03	Pancreatic cancer metastasis	No tumour	No tumour	No tumour	No tumour	0.9999	✓✓
G209-06-01	Non-disease associated	No tumour	No tumour	No tumour	No tumour	1.0000	✓✓
G209-06-02	Non-disease associated	No tumour	No tumour	No tumour	No tumour	0.9999	✓✓
G209-06-03	Non-disease associated	No tumour	Difficult to evaluate, normal liver (No tumour)	No tumour	No tumour/ Necrotic	0.9998	✓✓
G245-16-01	Non-disease associated	No tumour	No tumour	No tumour	No tumour	1.0000	✓✓
G245-16-02	Non-disease associated	No tumour	Difficult to evaluate, likely no tumour	No tumour	No tumour	1.0000	✓✓
G245-17-01	Colorectal cancer metastasis	Fibrotic, probably adenocarcinoma (tumour) right hand side	Fibrosis only	Fibrosis/ Tumour	No tumour	1.0000	××
G245-17-02	Colorectal cancer metastasis	Fibrotic, probably adenocarcinoma (tumour) lower right corner	Fibrosis only	Fibrosis/ Tumour	Tumour	1.0000	×✓
G278-08-01	Non-disease associated	Liver parenchyma, no tumour	Cannot confidently say what this is	No tumour/ Parenchyma	No tumour	1.0000	×✓
G278-08-02	Non-disease associated			No tumour	No tumour	1.0000	✓
G278-09-01	Colorectal cancer metastasis	Difficult, some necrosis? Adenocarcinoma (tumour)?	Difficult to evaluate, normal liver (No tumour)	Tumour/ Necrotic/ No tumour	No tumour	0.9990	×✓
G278-09-02	Colorectal cancer metastasis			Tumour	No tumour	1.0000	×
G278-09-03	Colorectal cancer metastasis			Tumour	No tumour	0.9999	×
G340-03-01	Colorectal cancer metastasis	Metastatic adenocarcinoma (tumour)	Likely [tumour] of some sort, can't determine origin	Tumour	Tumour	1.0000	✓✓
G340-03-02	Colorectal cancer metastasis	Metastatic adenocarcinoma (tumour)	Entire section shows tumour	Tumour	Tumour	0.9999	✓✓
G340-04	Non-disease associated	No tumour	Most material necrotic	No tumour/ Necrotic	No tumour/ Necrotic	0.9996	✓✓
G350-01	Non-disease associated	No tumour	Most material necrotic	No tumour/ Necrotic	No tumour/ Necrotic	0.9990	✓✓
G350-02-01	Colorectal cancer metastasis	Metastatic adenocarcinoma (tumour)	Likely [tumour] of some sort, can't determine origin	Tumour	Tumour	0.9876	✓✓
G350-02-02	Colorectal cancer metastasis	Metastatic adenocarcinoma (tumour)	Entire section shows tumour	Tumour	Tumour	1.0000	✓✓
G3113-01-01	Non-disease associated			No tumour	No tumour/ Necrotic	0.9799	✓✓
G3113-01-02	Non-disease associated	No tumour	Difficult to evaluate, likely no tumour	No tumour	No tumour/ Necrotic	0.9813	✓✓
G3113-06	Non-disease associated			No tumour	No tumour	1.0000	✓
G3113-07	Colorectal cancer metastasis			Tumour	Tumour	1.0000	✓
G3113-08	Colorectal cancer metastasis	Metastatic adenocarcinoma (tumour)	Entire section shows tumour	Tumour	Tumour	0.9992	✓✓

✓✓ Similar diagnosis from both pathologists matched by predicted cluster
×✓ Discordant diagnosis with one label matched by predicted cluster
×× Discordant diagnosis not matched by predicted cluster
✓ Nominal assessment matched by predicted cluster
× Nominal assessment not matched by predicted cluster
